# Black rice as the emerging functional food: bioactive compounds, therapeutic potential and industrial applications

**DOI:** 10.3389/fnut.2025.1705983

**Published:** 2025-10-29

**Authors:** Mavra Javed, Jawaria Jawid, Saira Zafar, Abdul Momin Rizwan Ahmad, Syed Hassan Bin Usman Shah, Umar Farooq, Juweria Abid

**Affiliations:** ^1^Department of Food Science and Human Nutrition, Michigan State University, East Lansing, MI, United States; ^2^Department of Nutrition and Dietetics, National University of Medical Sciences (NUMS), Rawalpindi, Pakistan; ^3^School of Public Health, Health Services Academy, Islamabad, Pakistan; ^4^Department of Human Nutrition and Dietetics, NUST School of Health Sciences, National University of Sciences & Technology (NUST), Islamabad, Pakistan; ^5^Department of Health Sciences, University of York, York, United Kingdom; ^6^The Kirby Institute, University of New South Wales, Sydney, NSW, Australia

**Keywords:** black rice, functional food, bioactive compounds, therapeutic potential, industrial applications, anthocyanins, phenolic acids, carotenoids

## Abstract

It is now widely understood that black rice, also known as *Oryza sativa* L., is a functional food and a nutritional powerhouse. In the past, it was known as forbidden rice. The current analytical viewpoints on recent breakthroughs in black rice research are investigated in this review paper. Topics covered include the bioactive chemicals of black rice, its potential therapeutic applications, and its applications in the food business. The fact that black rice includes bioactive compounds that have antioxidants, cardiovascular preventative properties, and anti-diabetic effects has been proven through clinical and metabolomic research. Additionally, this study addresses recent innovations in the gluten-free sector and packaging, as well as showing how processing methods influence bioactive substances. It is anticipated that the global market for black rice would be worth USD 15.14 billion by the year 2030. This reflects the growing demand among consumers for nutrient-dense superfoods. Taking this into consideration, there is a need for additional investigation into the true potential of black rice in the food and wellness business. This is necessary to ensure that this superfood may be utilized to the fullest extent of its potential in the future.

## 1 Introduction

Approximately 50% of the global population relies on rice (*Oryza sativa* L.) as a fundamental food source, serving as a primary energy provider. It is the paramount cereal crop utilized either directly as human sustenance or indirectly as livestock feed. Rice is cultivated in over 100 nations worldwide, extending from 45° S to 53° N latitudes. (1). It serves as the primary staple food in a minimum of 15 nations in Asia and the Pacific, 10 nations in Latin America and the Caribbean, one nation in North Africa, and seven nations in sub-Saharan Africa. Rice is ubiquitous, except for Antarctica (2). At least 50% of all rice produced is consumed within 10 miles of its cultivation site. Rice can be cultivated in any location and is the most productive cereal crop. A significant section of the global population allocates three-quarters of their income solely to rice. Approximately 95% of global rice production occurs in Asian countries (3). The terms “food” and “rice” are utilized interchangeably in various Asian languages. The United Nations General Assembly designated 2004 as the “International Year of Rice” (IYR) in acknowledgment of the significance of this crop. Rice ranks second in output, following corn. It can yield over 3,000 grains of rice from a single seed. In terms of human nutrition, rice accounts for around one-fifth of global caloric consumption. In Asia, almost 2 billion individuals derive 60–70% of their daily caloric intake from rice. Asian rice (*Oryza sativa* L.) serves as the primary cereal grain for 3 billion individuals globally (4). It constitutes up to 70% of caloric consumption in Southeast and South Asia and is fundamental to several civilizations in the region (FAOSTAT). Rice is extensively grown and consumed in East, Southeast, and South Asia. It arrived in Europe and America via European colonization (5). The precise origin of various colored rice cultivars remains ambiguous, however many trace their lineage to Asian regions, including India, China, and Thailand. These kinds have been farmed for millennia and are essential to the cuisine and culture of these places The significance of colored rice variants extends beyond their nutritional attributes. They significantly contribute to the diversification of agricultural production and the promotion of healthier, more balanced diets. Consuming these types can enhance the intake of antioxidants and other vital nutrients, hence promoting improved overall health and wellbeing (6). In Asian nations, particularly China and Indonesia, the general populace was prohibited from storing, cultivating, or consuming black rice during the imperial era without official authorization. it was exclusively reserved for royalty and aristocratic individuals, serving as a tribute food (7). In antiquity, it was believed that black rice could enhance the longevity and health of kings, and it was regarded as exceptionally rare and superior. Black rice, sometimes referred to as forbidden rice, imperial rice, king’s rice, purple rice, paradise rice, and treasured rice, is rich in antioxidants and micronutrients (8). In 2023, Asia-Pacific produced 82% of global black rice production of 1.2 million tons. China produced 470,000 tons, Indonesia 210,000, Thailand 150,000, and India 110,000. Over 350,000 hectares of organic black rice had been grown, up from 240,000 two years ago. Global conventional cultivation occupies 480,000 hectares. Black rice yields 3.2 tons per acre, while white rice yields 4.5 tons Black rice consumption per capita is 1.8 kg/year in industrialized markets and 0.9 kg/year in emerging regions (9). Online sales channels increased from 12% of market volumes in 2021 to 22% in 2023, and retail penetration reached 33 countries by late 2023. Retail prices for organic black rice average USD 4.50/kg, while conventional kinds cost USD 2.80/kg. Black rice is recognized for its antioxidants; lab study indicates anthocyanin amounts of 850 mg/kg, surpassing blueberries. Functional food manufacturers use black rice in over 420 snacks, porridges, rice blends, and ready-to-eat meals (10). Black rice belongs to the Oryza genus and has been cultivated in Asia for about 9,000 years (11). Scientific interest has surged in recent decades due to its extraordinary phytochemical density, which is 35 times greater than that of dazzling. The correlation between chronic diseases and postprandial metabolic syndrome (hyperglycemia/hyperlipidemia) is well documented, and black rice will contribute to the formulation of a suitable dietary strategy for these conditions (12).

Black rice is a nutrient-rich grain characterized by its balanced macronutrient composition and substantial micronutrient content. The United States Department of Agriculture (USDA) reports that 100 g of cooked black rice has around 356 kcal, 8.9 g of protein, 3.3 g of fat, 75.6 g of carbohydrates, and 2.2 g of dietary fiber. Recent analytical results confirm these findings, suggesting 7.5–9 g of protein per cooked cup, 34 g of carbohydrates, and 2–3 g of fiber, while also noting that black rice contains no cholesterol and has a low total fat content of approximately 1.5 g (13). In addition to macronutrients, black rice is rich in critical minerals, including iron (2.4 mg/100 g), zinc, manganese, calcium, phosphorus, and selenium. It also comprises essential B vitamins, including thiamine (B1), riboflavin (B2), niacin (B3), folate, and vitamin E in the form of tocopherols (14). The protein fraction contains all the necessary amino acids, including lysine and tryptophan, among others. The micronutrients in black rice render it a superior alternative to refined grains, thereby assisting in the maintenance of satiety, glycemic stability, and nutritional sufficiency (15). Black rice possesses a unique deep purple-black pericarp abundant in anthocyanins, specifically cyanidin-3-glucoside and peonidin-3-glucoside, concentrated in the aleurone layer. The economic potential is evidenced by a global market growth rate of 8.3% CAGR from 2025 to 2030 (16). The need to offer better food options for a growing population has been a major factor in the fast-increasing demand for plant-based goods around the world. The number of bioactive compounds in black rice products can be affected by the various post-harvest and industrial processing methods utilized, because of the varied processing circumstances. Its possible application as a functional grain to combat the worldwide epidemic of NCDs is the subject of ongoing research. Critics have pointed out the crop’s photochemistry and whole-food value, which means we need to update our data to account for new developments. To assess the health benefits and business potential of black rice, the review incorporates metabolomics, clinical, and food technology knowledge.

## 2 Nutritional profile and bioactive compounds of black rice

The weighted nutritional content data from databases and research studies on crops consistently indicates that black rice is a nutrient-dense food source. The historical nutritional analysis of the primary minerals and vitamins in rice highlights that black rice, alongside macronutrients, offers physiologically essential amino acids like lysine and tryptophan, B vitamins, vitamin E, and minerals including calcium, magnesium, phosphorus, and manganese, rendering it nutritionally superior to refined varieties (17).

A study indicates that the complete bran layer of black rice, which encompasses over 80% of the bioactive compounds, is responsible for its nutritional benefits. A quarter-cup serving of black rice contains around 5 g of protein and all 18 amino acids, including the essential amino acids tryptophan and lysine, which are relatively rare in most cereal grains containing approximately 3 g per quarter cup, of which roughly 75% is insoluble fiber, it serves as a substantial source of dietary fiber. Research indicates that a significant quantity of insoluble fiber is crucial for promoting satiety and facilitating cholesterol elimination (15).

The lipid composition of black rice mostly consists of oleic acid (36%), linoleic acid (34%), and palmitic acid (20%), which contribute to its nutritional and functional qualities (18). It possesses a notable mineral composition, with considerable quantities of iron (2.4 mg/100 g), zinc (3.6 mg/100 g), and selenium, which are essential for the functionality of antioxidant enzymes. Alongside these minerals and macronutrients, black rice contains all essential vitamins, including vitamin E as 2-Alphatocopherol, B-complex vitamins such as riboflavin (B2) and thiamin (B1), and 2-beta-carotene, which further augments the health-promoting attributes of black rice (19).

Recent research conducted by dietitians indicates that black rice contains a higher concentration of anthocyanins, specifically cyanidin 3 glucoside and peonidin 3 glucoside, than many berries (e.g., −456 mg/100 g), contributing to its functional value (15). Kum Akha black rice extracts inhibit the cytokines IL-6, IL-1β, and IL-18 via NLRP3 inflammasome pathways in immunological cell lines, highlighting the robust antioxidant and anti-inflammatory properties of these pigments. Moreover, phenolics having diverse health effects encompass rutin, quercetin, protocatechuic acid, ferulic acid, p-coumaric acid, and syringic acid. Tocopherol, tocotrienol, phytosterols, carotenoids (including lutein and zeaxanthin), 5-oryzanol with lipid-lowering properties, and 5-aminobutyric acid are supplementary bioactive compounds (20, 69).

### 2.1 Anthocyanins

Black rice belonged to *Oryza sativa* L. species, characterized by the presence of anthocyanins, specifically cyanidin-3-glucoside and peonidin-3-glucoside, exhibit a dark-purple hue and possess significant antioxidant, anti-inflammatory, anti-diabetic, and lipid-modulating properties (21). Dietary supplementation with 2.5–5% germinated waxy black rice (GWBR) in streptozotocin-induced diabetic rats over an 8-week period led to a reduction in the transcription factors hepatocyte nuclear factors HNF-1α, HNF-1β, and HNF-4α (22).

### 2.2 Phenolic acids and flavonoids

The most prominent phenolic acids include ferulic, p-coumaric, protocatechuic, and syringic acids, which provide significant antioxidative and anti-inflammatory support. LC–MS profiling shows that ferulic acid comprises ~45% of the sum free phenolics and has ORAC values of 20,000 and more umol TE/100 g, that is, six times as much as brown rice. These phenolic acids impede the activation of NF κB triggered by ROS and enhance the levels of endogenous antioxidant proteins (SOD, catalase), so bolstering cellular defense mechanisms (23).

### 2.3 γ-Aminobutyric acid (GABA)

GABA is a non-protein amino acid neuromodulator that rapidly increases during germination, reaching concentrations of up to 300 mg/kg in sprouted grains. The rise is associated with enhanced glucometabolic regulation and neurocognitive benefits, including improved memory and mood, noted in animal studies and limited human cohorts (24) (Table 1).

**Table 1:** Key bioactive compounds in black rice.

Compound class	Specific compounds	Concentration	Location
Anthocyanin	Cyanidin-3-O-glucoside	2568.63 mg/100 g (bran)	Aleurone layer (75)
	Peonidin-3-O-glucoside	95.46 mg/100 g	
Phenolic acids	Ferulic acid	96.97 μg/g (bran)	Bran/pericarp (76)
	p-Coumaric acid	50.27 μg/g	
Carotenoids	Lutein, Zeaxanthin	0.87–1.23 mg/100 g	Endosperm (77)
γ-Aminobutyric Acid (GABA)	–	25.6 mg/100 g (germinated)	Germ (78, 79)

### 2.4 γ-Oryzanol and tocopherols

Oryzanol, a combination of ferulic acid esters, along with tocopherols (vitamin E constituents), is prevalent in the bran layer, with an estimated concentration of 100–200 mg/kg. These lipophilic antioxidants exert an antioxidant effect by diminishing LDL levels and cardiovascular disease through the inhibition of cholesterol absorption and the elevation of HDL levels (25).

### 2.5 Dietary fiber and resistant starch

While not conventionally categorized as phytochemicals, dietary fiber (~2–3 g/100 g) and resistant starch (~15% in high-amylose variants) are essential for gastrointestinal health, glycemic regulation, and satiety. These interact with bioactive compounds to extend glucose release and enhance microbial diversity (26).

## 3 Mechanism and clinical evidence of therapeutic potential

### 3.1 Antidiabetic effect

The antidiabetic effects are primarily attributed to the anthocyanin-mediated inhibition of carbohydrate-hydrolyzing enzymes (alpha amylase and alpha glucosidase) and the suppression of intestinal glucose transporters (SGLT1 and GLUT2), which collectively mitigate postprandial glucose spikes by %%30–35%. The quantity of resistant starch in specific varieties of black rice enhances these effects by decelerating carbohydrate digestion and augmenting insulin sensitivity (27). The IC50 value for α-glucosidase is noticeably lower than that for α-amylase, suggesting that the phenolic chemicals found in black rice are more efficient against this enzyme (28). Black rice’s high polyphenol content interacts with digestive enzyme molecules through non-covalent interactions, altering their structure and function. The hypoglycemics properties of polyphenols make them an effective adjuvant in the treatment of diabetes (27). Evidence for a role for black rice extracts (BRE) in glucose homeostasis was shown in insulin-resistant rats given a high-fat diet, where it significantly decreased plasma glucose and improved lipid profiles (29). Previous research suggests that diabetic rats’ glucose homeostasis and blood glucose levels were improved after 8 weeks of BRE administration, likely because of improved glucose regulation and prevention of abnormal glucose production. Furthermore, γ-oryzanol reduced adipocyte lipid buildup, and obese mice showed an improvement in lipid metabolism after consuming black rice with large embryos (30).

### 3.2 Dyslipidemia management

In the context of cholesterol management, anthocyanins found in black rice inhibit intestinal cholesterol absorption by downregulating Niemann-Pick C1-like 1 (NPC1L1) transporters and enhance fecal sterol excretion by upregulating ATP-binding cassette subfamily G member 5/ATP-binding cassette subfamily G member 8 (ABCG5/ABCG8). Animal models have demonstrated a 22% reduction in LDL cholesterol due to these molecular mechanisms, and beneficial lipid ratios have been observed in preliminary human studies (31). The reduction of LDL cholesterol levels and the preservation of endothelium health relieve the interplay of dietary fiber, phytosterols, and anthocyanins. Owing to its antioxidant properties, the consumption of whole grains, especially black rice, is associated with a reduced risk of cardiovascular diseases (25). Researchers at Harvard School of Public Health have predicted that substituting approximately two servings of white rice per week with an equivalent quantity of black rice could reduce the risk of diabetes by 16%. Insulin resistance is intricately linked to non-alcoholic fatty liver disease. Multiple studies indicate that natural anthocyanins are powerful antioxidants linked to prevention of diabetes. Jang et al. (72) proposed that black rice containing C3G may mitigate the risk of hepatic fat buildup and enhance insulin resistance. A clinical study published in the “American Journal of Clinical Nutrition” examined the effects of key flavonoid groups and determined that anthocyanins are the only category significantly linked to a reduced risk of Type II Diabetes (73). Scientific studies conducted on rats, with dosages equivalent to 120 mg of C3G for humans, demonstrated that C3G inhibited lipid peroxidation (cell membrane damage resulting in cell death), enhanced superoxide dismutase activity (the body’s antioxidant defense mechanism), and exhibited a hypoglycemic effect (reduction of blood sugar levels) over an eight-week period (74). C3G lowers blood glucose levels and is thus regarded as having significant anti-diabetic properties (mitigation of diabetic development) linked to metabolic syndrome (antioxidant and anti-inflammatory actions).

### 3.3 Cardiovascular health

The cardiovascular benefit extends beyond cholesterol management. The mechanism of action involves the stimulation of endothelial nitric oxide synthase (eNOS) via the PI3K/Akt pathway, leading to augmented vasodilation and a 12% increase in flow-mediated dilation, indicative of endothelial function (32). Neuroprotective benefits are also delineated in both preclinical and clinical research. Anthocyanins in black rice has antioxidative properties as they neutralize reactive oxygen species (ROS), impede NF-kB translocation, and augment the Nrf2/HO-1 pathways to mitigate oxidative damage in the brain. The amalgamation of these advantages underpins the potential and applications of black rice as a versatile grain with designated roles in the regulation of metabolic, cardiovascular, and cognitive health (33). Anthocyanins in black rice inhibit the enzymes α-amylase and α-glucosidase, hence diminishing starch hydrolysis. In clinical trials, consumption of black rice reduced postprandial glucose increases by 30–35% in comparison to white rice A 2025 metabolomics analysis validated that anthocyanins interfere with glucose absorption through SGLT1/GLUT2 transporters in enterocytes. Resistant starch (up to 15% in high-amylose types) further mitigates glycemic reactions (34). A meta-analysis indicates that the anthocyanins in black rice function as amylase and glucosidase inhibitors, reducing postprandial glycemic and lipid responses by obstructing bile acid binding and cholesterol micelle formation. Clinical studies demonstrated reduced postprandial glucose and lipid levels, along with enhanced antioxidant status after eating black rice, support these mechanisms (35).

### 3.4 Oxidative stress management

The efficacy of black rice anthocyanins in neutralizing free radicals and mitigating oxidative stress has been evidenced in both animal models and cell cultures, establishing them as principal antioxidants. The elevated anthocyanin concentration in Kum Akha’s butanol fraction significantly decreased pro-inflammatory cytokine production by THP-1 and RAW264.7 macrophages. Moreover, anthocyanins demonstrated the ability to mitigate cytokine storms in lung cell models induced by the SARS-CoV-2 spike protein, potentially affecting respiratory health.

Recent *in vitro* studies have demonstrated that anthocyanin fractions from black rice bran protect neuronal cells from oxidative and endoplasmic stress induced by amyloid-beta, the primary contributor to Alzheimer’s disease. These data indicate neuroprotective potential, which may inform future Alzheimer’s therapy strategies. Lutein and zeaxanthin protect the retinal pigment epithelium from UV damage (0.87–1.23 mg/100 g). Hepatic protective factors encompass modified hepatic fibrosis and the control of TGF-B1 signaling (36).

The health-promoting benefits of black rice are primarily attributed to its elevated levels of anthocyanins, particularly cyanidin 3 glucoside (C3G) and peonidin 3 glucoside (P3G). The bran of Kum Akha black rice possesses an anthocyanin-rich fraction (KA1 P1) that markedly suppresses the activation of the NLRP3 inflammasome (37). This diminishes the expression of NLRP3 and subsequent cytokines (IL-1β and IL-18) in the lung tissues of human macrophage cell models and mouse subjects. The outcome represents a unique mechanism potentially applicable in the treatment of viral respiratory illnesses characterized by a hyper-inflammatory response. Extracts of black rice germ and bran, high in C3G and P3G, markedly inhibited NF and inflammatory gene expression of spike-protein S1 in A549 lung cells and THP-1 macrophages (38).

### 3.5 Anti-cancer properties

**Figure 1:**
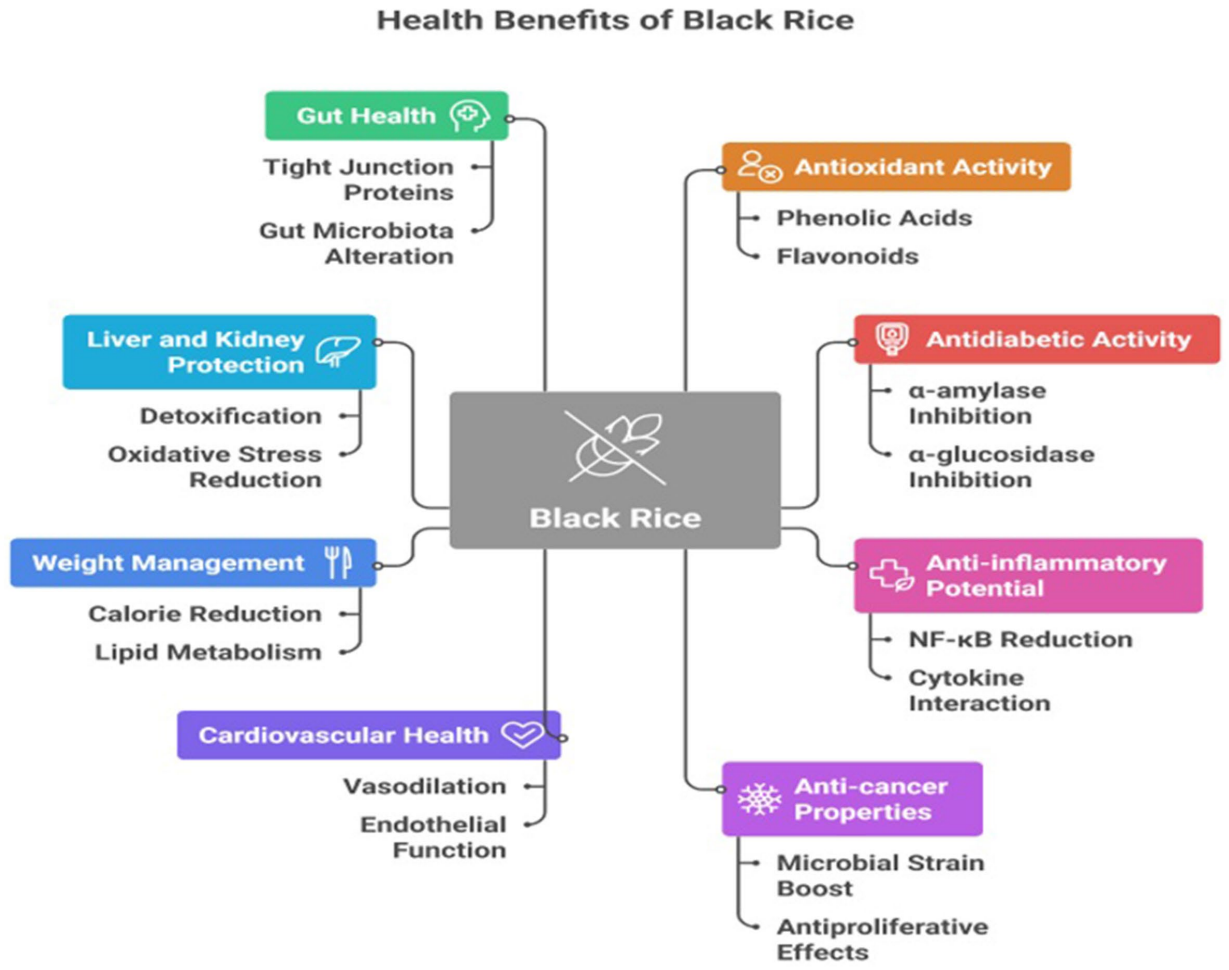
Health benefits with possible mechanism of black rice.

Cancer is one of the most lethal diseases in the present context, mostly resulting from oxidative stress in the body. Increased oxidative stress results in a higher production of reactive oxygen species and reactive nitrogen species, which disrupt genetic material in cells and cause damage that can lead to carcinogenesis (39). Black rice, rich in antioxidants and fiber, has the potential to reduce oxidative stress and hence carcinogenesis. Kum Phayao bran exhibited chemo-preventive properties in rats induced with hepatocarcinogenesis by Aflatoxin B1. The antimetastatic properties of peonidin 3-glucoside and cyanidin 3-glucoside, two main anthocyanins derived from black rice (*Oryza sativa L. indica*), were demonstrated molecularly through a significant suppression of SKHep-1 cell invasion and motility. The expression of matrix metalloproteinase-9 (MMP-9) and urokinase type plasminogen activator (u-PA) was shown to be decreased in conjunction with this impact. Like cyanidin 3-glucoside, peonidin 3-glucoside inhibited AP-1 nuclear translocation and DNA binding activity (20, 71) In addition, SCC-4, Huh-7, and HeLa cells were inhibited in their invasion by these chemicals. In conclusion, the anthocyanins (OAs) derived from Origanum sativa *L. indica* were shown to suppress the *in vivo* development of SKHep-1 cells (40) (Figure 1).

## 4 Applications in the food industry

### 4.1 Effects of processing on nutrient and phytochemical integrity

The processing procedures significantly influence the preservation or degradation of bioactive chemicals in black rice. A thorough metabolomics study conducted in recent years evaluated the changes in metabolites of black rice following milling, germination, and exposure to high temperature and high pressure. The findings indicated that germination enhanced the levels of GABA, γ-oryzanol, and phenolic compounds, whereas milling led to a reduction in anthocyanins (35). Pressure heat treatment was associated with microbiological safety; nevertheless, it may not affect metabolite levels without impacting germination, which is essential when employing germination alongside high-pressure heat treatment for processing functionally valuable food (41). Similar studies on anthocyanin degradation during the milling of purple glutinous rice indicated that polishing over 4% resulted in a reduction of 50–90% of anthocyanins, with nearly complete loss occurring at 9% polishing or higher (42). Post-harvest sprouting, fermentation, and UV-C irradiation may partially enhance or elevate bioactive levels. Moreover, enzymatic acylation methods enhanced antioxidant capacity and anthocyanin stability, indicating prospective avenues for the advancement of more stable functional constituent (43, 44).

Despite the numerous benefits of black rice, certain processing methods may affect its bioactive compounds. Milling has been shown to eliminate more than 85% of total phenolics and 74% of anthocyanins when utilized to preserve the bran layer. A thermal processing method that effectively reduces hazardous cyanidin-3-lucoside levels by 40–60% is high-temperature drying. Steaming seems to exert a lesser effect on the loss of nutritional content compared to boiling, which causes a 15–30% reduction in water-soluble anthocyanins (45). Nonetheless, several processing techniques can enhance the bioactivity of black rice. The enzymatic activation mechanism during germination elevates GABA concentration, raising it sevenfold, while concurrently generating a substantial amount of phenolic acid. Fermentation utilizing microbial strains, such as Lactobacillus species, which decompose complex compounds during metabolism, might enhance the bioavailability of anthocyanins. High-pressure processing (HTP) at 115 °C for 20 min not only stabilizes certain heat-stable flavonoids, such as malvidin derivatives, but also seems to be an effective way for preserving the functional properties of black rice during industrial processing (46) (Table 2).

**Table 2:** Processing effects on key metabolites.

Processing method	Impact on metabolites	Change vs. Raw black rice
Milling	Decreased phenolic acids, anthocyanins, and dietary fiber	−73.9% anthocyanins (80)
Germination (42 h, 30 °C)	Increased GABA, organic acids, amino acids; Decreased polyketides	+65.7% GABA (80, 81)
HTP (115 °C, 20 min)	Increased Heat-stable flavonoids; Increased β-oxidized fatty acids	+22% quercetin derivatives (80)
Fermentation	Increased accessible anthocyanins; Increased peptide antioxidants	+40% antioxidant capacity (81)

### 4.2 Functional food products and sensory considerations

Black rice has diverse applications in the culinary industry, drawing from both traditional and contemporary products. Utilizing 10–40% black rice flour in place of wheat flour for bread baking has demonstrated a reduction in the glycemic index by around 25%, while enhancing antioxidant capacity, so rendering the flour both functional and healthy (47). The beverage industry recognizes that fermented black rice drinks, such as koji, contain a significant gamma-aminobutyric acid (GABA) concentration of up to 156 mg per 100 g, perhaps associated with neuroprotective properties (48, 70). The incorporation of anthocyanin-enriched black rice extracts into the coatings of extruded snacks has been suggested as a method to prolong their shelf life and offer a pH-sensitive color change indicative of spoilage (49, 50).

#### 4.2.1 Bread and baked products

Incorporating anthocyanins (with up to 85% retention of germinated wheat) and substituting black rice flour (10–20%) enhances the nutritional profile of bread by (51). This insoluble fiber (2.8 g/100 g) enhances superior dough and offers prebiotic benefits. It also assert that optimal sensory attributes are attained with a 25–30% substitution; nevertheless, elevated ratios, particularly with hydrocolloids such as xanthan gum, are necessary to maintain crumb structure (52). Commercial innovations during this period encompass BRRI’s high-fiber loaves, designed to enhance metabolic health, and Rani Foods World’s anthocyanin-enriched breakfast bread products (53, 54)(Table 1).

#### 4.2.2 Breakfast cereals and snacks

The resistant starch in extracted black rice grains (up to 15% in high-amylose varieties) retards the release of glucose during digestion. Processing at temperatures up to 115 °C can maintain anthocyanin retention, achieving levels of 70–80% in puff snacks. Food waste will be diminished through the visual package spoiling assessment enabled by anthocyanins, which alter color in reaction to pH fluctuations (29).

#### 4.2.3 Fermented functional drinks

The γ-aminobutyric acid (GABA) content (156 mg/100 g) in traditional Asian koji-fermented black rice beverages possesses neuroprotective qualities. The fermentation of the Lactobacillus strain enhances anthocyanin absorption by 40% in terms of antioxidant capacity. Probiotic smoothies with black rice bran and plant-based yogurts exemplify contemporary approaches aimed at enhancing the gut-brain axis (55).

#### 4.2.4 Anthocyanin-infused beverages

Black rice extracts serve as natural colorants in functional beverages, including fizzy drinks and functional juices, as substitutes for artificial colors. Anthocyanins, such as cyanidin-3-O-glucoside, are well suited for acidic soft beverages like black rice colas due to their stability within the pH range of 2.5–4.0. The rice beverages exhibited the lowest results in the ORAC experiment, with measurements ranging from 1,845.7 to 6,103.5 μM Trolox/L (56).

#### 4.2.5 Gluten-free pasta

Incorporating 20–35% black rice flour in place of wheat pasta flour enhances dietary fiber content (3 g per quarter-cup) and reduces the predicted glycemic index by 20 points. The integrity of protein matrices is enhanced by the phenols in the aleurone layer (ferulic acid: 96.97 g/g), which inhibits the texture from being overdone. Commercial food products providing cardiovascular protection, such as the Italian produced black rice linguine, are targeted toward celiac consumers (57).

#### 4.2.6 Instant noodles and Asian staples

The incorporation of black rice flour (15–25) into instant noodles enhances their nutritional profile and diminishes oil absorption during frying by 18%, attributable to its elevated amylose concentration. Thai purple rice noodles, which contain 0.87 mg of lutein and zeaxanthin per 100 g, have traditionally leveraged the adhesive properties of glutinous kinds to enhance their culinary applications, thereby promoting eye health. The Optimized Black Rice-based Instant Idli Mix (BRIIP) comprises 34% black rice, has 402.33 mg GAE/100 g of phenolic compounds, and demonstrates microbiological stability during storage (58).

## 5 Emerging clinical evidence and human trials

Black rice bran anthocyanin extracts serve as a pH-sensitive indicator in chitosan-based films. As the pH increases, these intelligent labels transition from purple to pink or yellow, indicating that protein-rich foods such as fish and poultry are on the verge of spoilage. Recent advancements utilize ZnO nanoparticles to enhance antibacterial capabilities and prolong the shelf life of beef by 30% (59).

It is shown that 80% of the anthocyanin remained after simulated digestion. This microencapsulated black rice bran (20–50 μm particles) excels in dietary supplements. Clinical trials have validated the efficacy of XIPHIAS Superfoods’ 500 mg/dose anthocyanin capsules as glycemic regulators, leading to a reduction of glucose levels by 50% postprandially (60).

While there is fewer human data corroborating the health benefits of black rice, existing evidence indicates that it may assist in reducing body fat and managing neurocognitive and cardiometabolic problems. In a 12-week randomized, double-blind, placebo-controlled trial, 88 obese postmenopausal Korean women exhibited a significant reduction in trunk fat, total fat mass, and total body fat %age, while body weight and BMI remained unchanged after dietary intervention with black rice extract compared to placebo (61). Conversely, purified black rice anthocyanins administered at 320 mg/day for 28 days did not affect LDL cholesterol, total or HDL cholesterol, triglycerides, apolipoproteins, or glycemic indicators in a randomized, placebo-controlled, double-blind, three-arm crossover research including 52 hyperlipidemic adults. This aligns with the idea that a brief intervention or singular extract preparation is inadequately effective (62).

Acute intake of pigmented rice for a duration of 30–180 min elevated plasma antioxidant capacity, whereas prolonged consumption (12–24 weeks) yielded modest yet significant reductions in body weight and fasting glucose levels, as reported in a systematic review and meta-analysis of 17 randomized controlled trials (63). Furthermore, sustained interventions revealed that 2 h post-consumption of 100 g of cooked black rice, there was an elevation in the levels of total phenolics and flavonoids in the bloodstream, alongside an increase in antioxidant activity, indicative of rapid bioavailability. A daily dosage of 1 g of germinated black rice extract significantly improved response times in working memory tasks, enhanced attentional control, and reduced the activity of plasma acetylcholinesterase and monoamine oxidase, which are associated with cognitive enhancement, as evidenced by an 8-week randomized, double-blind, placebo-controlled trial conducted on healthy Thai adults aged 44–56 years (64). Alongside facilitating further clinical research to ascertain a rigorously effective dosage, prepare the extract source, and evaluate long-term outcomes through extensive, longitudinal, and standardized trials, these human studies collectively illustrate the potential of black rice in body composition, cardiometabolic processes, and neurocognition.

The commercialization of items derived from black rice is an additional factor influencing market dynamics. The global market for organic and powdered black rice is projected to attain USD 15.14 billion by 2030, driven by substantial demand. While black rice is accessible in China, its market is growing in the United States (NPR). Premium commercial foods encompass nutraceutical offerings such as Superfoods’ black rice extract capsules for blood sugar regulation, functional cereals exemplified by anthocyanin-rich breakfast mixes from Rani Foods and World Foods, and high-quality ingredients like Lotus Foods’ heirloom black rice utilized in ready-to-eat meal products (65).

Reasons for black rice’s rapid ascendance in the worldwide functional food sector include its advantageous anthocyanin and phytochemical constituents, along with its versatile characteristics. Forecasts indicate that the global market for functional foods and beverages is projected to increase from a value of US $320 billion in 2024 to over US $384 billion by 2028, with plant-based inventions such as black rice being a major reason to this expansion (66). Consumers are demanding clean-label food products, prompting research into the incorporation of anthocyanin-rich black rice in plant-based milk, antioxidant-enriched juice, snack bars, and gluten-free pasta. Cultural authenticity is being combined with nutritious value to target nutrient-conscious consumers in Europe and North America, exemplified as India’s GI-protected Chak-Hao rice (67).

## 6 Research gap and future directions

### 6.1 Clinical and human health gaps

Numerous essential information deficiencies hinder the effective incorporation of black rice into clinical practice and extensive commercial food networks, notwithstanding the current increase in academic and market interest in this grain. Currently, human outcomes are limited by sample size and the consistency of outcome measurements, particularly regarding cardiovascular health, metabolic syndrome, diabetes, and cognitive decline. However, standardized, adequately powered, long-duration treatments contrasting whole grain consumption with isolated extracts are necessary. The second issue is the ambiguous definition of dosage responses: intact anthocyanins in plasma seldom exceed 5%, and the dietary and oral doses of anthocyanins in animal studies (sometimes surpassing 200 mg/kg) are impractical in the context of human consumption (68). Consequently, investigations into pharmacokinetics and bioavailability must be conducted on human subjects.

### 6.2 Industrial and commercialized challenges

Regarding the industrial aspect, challenges related to scalability, cost, compatibility with sensory attributes, and storage persist, despite progress in bioactive retention via germination and subsequent high temperature/pressure (HTP) treatments. We must focus on customer research and life-cycle studies to impact future commercial development. Low yield, extreme photosensitivity, a lengthy vegetative phase, and a tall stature that creates loading are some of the inherent unfavorable qualities that make it unpopular among farmers. Nutrient-enriched black rice is out of reach for the average person due to its high price and low production. Securing a consistent supply of raw materials necessitates agronomic research to elucidate genotype-environment interactions, encompassing altitude, soil type, and environmental stresses, to ascertain anthocyanin accumulation, which peaks at 15–21 days post-flowering (1). Nonetheless, the imperative for sustainability remains the process of black rice production requires water-intensive solutions, and the utilization of bran must adhere to circular economy principles to reduce byproducts and environmental impact.

### 6.3 Bioavailability and accessibility

Investigating the bioavailability and bioaccessibility of black rice bioactive composites should be the focus of future studies so that their therapeutic potential can be better understood. Modern analytical methods, such proteomics and metabolomics, can reveal novel bioactive composites and the processes by which they work. Longitudinal clinical trials are also necessary to prove that black rice is healthy and to provide evidence-based dietary recommendations. Examining the components of black rice in conjunction with one another, rather than in isolation, will provide light on its health benefits. For black rice to be more useful and consistent in food products, innovative processing methods need to be mass-produced. This includes improving extraction methods for bioactive compounds and extending shelf life to prevent rancidity. Incorporating into medications, functional foods, and supplements is one of the encouraging approaches to tackling global health challenges. Finally, public health initiatives can promote the use of black rice in regular diets by highlighting its nutritional and therapeutic advantages. Following these future approaches can turn black rice from an agricultural byproduct into an essential part of preventative and therapeutic nutrition.

## 7 Conclusion

Ultimately, black rice has many practical and health-related applications; it is a functional food that is culturally significant, nutrient-rich, and supported by science. It has a lot of minerals, resistant starch, phenolic compounds, dietary fiber, and anthocyanins, mostly in the form of cyanidin-3-glucoside. These ingredients have the real potential to fight obesity, help regulate blood sugar, boost heart health, enhance brain function, decrease inflammation, and lessen oxidative stress. The technical feasibility of bios has already been scientifically and mechanistically verified in animal models, even though human studies remain in the nascent phases. The metabolic and cognitive benefits are supported by this; nevertheless, there is some diversity in the therapeutic outcomes caused by factors such as extract type, dosage, and duration. Beverages, baked goods, gluten-free and functional snacks, colorants, and novel packaging are just a few areas where black rice continues to be an innovative ingredient in the food industry. Rising consumer interest is driving increased integration, but progress requires comprehensive clinical studies, clarity of bioavailability data, scalable manufacturing methods, sustainable production practices, consistent agronomic reproducibility, compliance with regulatory standards, and sustainable production practices. This study shows that black rice is a real thing and has been proven by science and industry.

## Abbreviations

3T3-L1: Mouse embryonic fibroblast cell line
A549: Human lung carcinoma cell line
ABCG5: ATP-binding cassette sub-family G member 5
ABCG8: ATP-binding cassette sub-family G member 8
AP-1: Activator Protein 1
BMI: Body Mass Index
BPT: Black Rice Genotype BPT 2841
BRE: Black Rice Extract
BRIIP: Black Rice-based Instant Idli Mix
BRRI: Bangladesh Rice Research Institute
C3G: Cyanidin-3-glucoside
CAGR: Compound Annual Growth Rate
CRC: Colorectal Cancer
DNA: Deoxyribonucleic Acid
FAOSTAT: Food and Agriculture Organization Statistical Database
GABA: Gamma-Aminobutyric Acid
GAE: Gallic Acid Equivalent
GI: Glycaemic Index
GLUT2: Glucose Transporter 2
HDL: High-Density Lipoprotein
HO-1: Heme Oxygenase-1
HTP: High-Temperature/Pressure Processing
IC50: Half Maxim
